# A road map for in vivo evolution experiments with blood‐borne parasitic microbes

**DOI:** 10.1111/1755-0998.13649

**Published:** 2022-06-06

**Authors:** Ruth Rodríguez‐Pastor, Yarden Shafran, Nadav Knossow, Ricardo Gutiérrez, Shimon Harrus, Luis Zaman, Richard E. Lenski, Jeffrey E. Barrick, Hadas Hawlena

**Affiliations:** ^1^ Jacob Blaustein Center for Scientific Cooperation, The Jacob Blaustein Institutes for Desert Research Ben‐Gurion University of the Negev Midreshet Ben‐Gurion Israel; ^2^ Mitrani Department of Desert Ecology, Swiss Institute for Dryland Environmental and Energy Research, The Jacob Blaustein Institutes for Desert Research Ben‐Gurion University of the Negev Midreshet Ben‐Gurion Israel; ^3^ Koret School of Veterinary Medicine, Faculty of Agricultural, Nutritional and Environmental Sciences The Hebrew University of Jerusalem Rehovot Israel; ^4^ Department of Ecology and Evolutionary Biology, The Center for the Study of Complex Systems (CSCS) University of Michigan Ann Arbor Michigan USA; ^5^ Department of Microbiology and Molecular Genetics Michigan State University East Lansing Michigan USA; ^6^ Department of Molecular Biosciences The University of Texas Austin Austin Texas USA

**Keywords:** *Bartonella* spp., experimental evolution, host–parasite adaptation, in vivo experiments, microbial pathogens, population bottlenecks, sequential passages

## Abstract

Laboratory experiments in which blood‐borne parasitic microbes evolve in their animal hosts offer an opportunity to study parasite evolution and adaptation in real time and under natural settings. The main challenge of these experiments is to establish a protocol that is both practical over multiple passages and accurately reflects natural transmission scenarios and mechanisms. We provide a guide to the steps that should be considered when designing such a protocol, and we demonstrate its use via a case study. We highlight the importance of choosing suitable ancestral genotypes, treatments, number of replicates per treatment, types of negative controls, dependent variables, covariates, and the timing of checkpoints for the experimental design. We also recommend specific preliminary experiments to determine effective methods for parasite quantification, transmission, and preservation. Although these methodological considerations are technical, they also often have conceptual implications. To this end, we encourage other researchers to design and conduct in vivo evolution experiments with blood‐borne parasitic microbes, despite the challenges that the work entails.

## 1 IN VIVO EVOLUTION EXPERIMENTS ARE ESSENTIAL FOR UNDERSTANDING PARASITE RESPONSES TO SELECTIVE PRESSURES IN NATURE

Understanding how populations of parasites, including pathogens, respond to selective pressures in nature is a challenge because it requires approaches that incorporate both ecology and evolution. One experimental approach is to sequentially propagate parasites through various animal or plant host environments. Evolved populations can then be compared to the ancestor. In particular, genetic and phenotypic changes that evolve can be assessed to understand whether and how the parasites have adapted, and to test alternative evolutionary patterns and mechanisms (e.g., virulence and resistance evolution). In vitro parasite evolution experiments that are conducted outside of host organisms, such as in cultures of host cells or in defined media with only the parasite present, have been used to observe evolution in replicate populations under controlled environmental conditions (Hall et al., 2012; Mehta et al., 2018; Tait‐Kamradt et al., 2000). However, often, the outcomes of such experiments do not reflect the complexity typical of natural conditions (Ebert, 1998). For example, Hernandez and Koskella (2019) found that the evolution of bacterial pathogens resistant to lytic bacteriophages was less common when a bacterium‐phage pair was grown on tomato plants than when the same partners were propagated in vitro on either artificial medium or tomato leaf apoplasts.

More generally*,* when one seeks to better understand parasite evolution, in vivo environments are preferable to in vitro environments because host organisms better emulate the natural environment of the parasites. For instance, the availability of resources for the parasites is often lower in vivo than in vivo, the environmental conditions (e.g., temperature, pH, and osmotic condition) are typically less stable, and the parasites may face competition from co‐occurring genotypes or species. Furthermore, an in vivo experiment can use a host population that forces the parasite to deal with host resistance mechanisms (Hoang et al., 2016). These differences between in vivo and in vitro experimental setups are likely to produce disparate evolutionary responses (Hindré et al., 2012). Thus, in vivo experiments can more accurately reflect the complexity and suite of selection pressures that act on microorganisms. In vivo experiments face many challenges, as we will discuss, but with a careful design one can overcome these challenges by manipulating parameters of particular interest (e.g., host strain) while controlling other variables (e.g., host diet) and study the effects of target parameters on in vivo evolution in detail (e.g., effects of microbial coinfection and immune response). In short, these experiments offer new opportunities to study parasite evolution and adaptation in real time and under more natural settings.

To date, in vivo evolution experiments have provided insights into parasite virulence (Alizon et al., 2013; Ben‐Ami et al., 2011), host‐microbe associations (Brockhurst & Koskella, 2013; Hart et al., 2019; Robinson et al., 2018), local adaptation (Agha et al., 2018; Batstone et al., 2020; Giraud et al., 2017), genomic evolution (Schmitt et al., 2020), and the generation and maintenance of host genetic diversity (González et al., 2019; Kubinak et al., 2015). They have also been used in the development of live attenuated vaccines against a number of viral and bacterial diseases (e.g., Koprowski et al., 1952). Importantly, depending on the study goals, these studies have used a variety of model organisms, ranging from laboratory‐selected and engineered strains in mutant hosts to natural isolates in wild hosts.

## 2 BLOOD‐BORNE PARASITIC MICROBES AS SUBJECTS FOR EVOLUTION EXPERIMENTS

Bacteria and viruses are particularly suitable subjects for in vivo evolution experiments owing to their small genomes, short generation times, high mutation rates, and large population sizes, which allow evolutionary changes to be observed as they happen (Van den Bergh et al., 2018). Moreover, the ease with which microbes, their environments, or both can be manipulated broadens the scope of questions that can be addressed through experimentation (Van den Bergh et al., 2018). For example, one can manipulate factors such as the host immune response (Cornwall et al., 2018) and resource availability (Karve et al., 2016) as well as parasite interactions (Hart et al., 2019), recombination (Cooper, 2007), mutation rates (Loh et al., 2010; Sprouffske et al., 2018), and genetic relatedness (Bashey et al., 2007). Perhaps the most important feature of microbes in the context of evolution experiments is their ability to be revived after long‐term storage in a nonevolving state, which enables a sort of “time travel” (Lenski & Travisano, 1994; Van den Bergh et al., 2018).

Blood‐borne parasitic microbes constitute an important group of pathogens that can be studied by performing in vivo evolution experiments. Importantly, they can be sampled without terminating the host's life by taking a blood sample, and they can be passed between hosts by inoculating new individuals. Moreover, the knowledge gained through such experiments often has implications for human health. Indeed, blood‐borne parasitic bacteria (*Anaplasma*, *Bartonella*, *Borrelia*, *Brucella*, *Coxiella*, *Francisella*, *Rickettsia*, *Yersinia*; e.g., Rejmanek et al., 2012), protozoa (*Leishmania*, *Plasmodium*, *Trypanosoma*; e.g., Sinha et al., 2018), and viruses (Chikungunya virus, Dengue virus, Eastern equine encephalitis, Japanese encephalitis virus, Venezuelan equine encephalitis virus, West Nile virus, Zika virus, Yellow fever virus; e.g., Patil et al., 2012) have long been studied using serial passage experiments in vertebrate hosts. These studies have contributed to our understanding of the pathogens' dynamics, virulence, morphological variation, gene‐expression variation, attenuation (for vaccine development), and adaptation to their hosts (Davies et al., 2011; Deardorff et al., 2011; Tian et al., 2018).

## 3 CHALLENGES OF IN VIVO EVOLUTION EXPERIMENTS WITH BLOOD‐BORNE PARASITIC MICROBES

Despite their importance, effective in vivo experimental studies of parasites entail many challenges. Rearing host organisms and transferring parasites between them are costly and labor intensive; as a consequence, such experiments are generally constrained by small sample sizes, limited timescales, and the potential for misinterpretation of the underlying processes (Kawecki et al., 2012). Moreover, some choices made in experimental design may inadvertently create unintended biases. For example, low infection rates might cause severe bottlenecks (reduction in parasite population size and concomitant loss of genetic diversity), potentially impeding the parasite's evolutionary response to the treatment (e.g., Nilsson et al., 2005). Thus, the specific experimental design and exact methodology play important roles in realizing the potential benefits of in vivo evolution experiments.

Blood‐borne parasitic microbes present additional complications. Many of these microbes are nonculturable, difficult to detect, isolate, and quantify, and can only grow on nonselective media (e.g., blood or chocolate agar) or under modified atmospheres (e.g., capnophilic or microaerophilic conditions; see Ahmed, 2014). In addition, isolation of microbes from blood and inoculating with infected blood may cause bottlenecks and unintended selection, and these procedures may also inadvertently introduce immune factors, reaction inhibitors, and contaminants (e.g., Jones et al., 1993). Thus, the main challenge when designing an in vivo evolution experiment with a blood‐borne parasitic microbe is to establish a transfer protocol that it is practical to maintain for multiple host passages while matching natural transmission mechanisms as closely as possible.

## 4 OUR STUDY'S GOAL AND APPROACH

**FIGURE 1:**
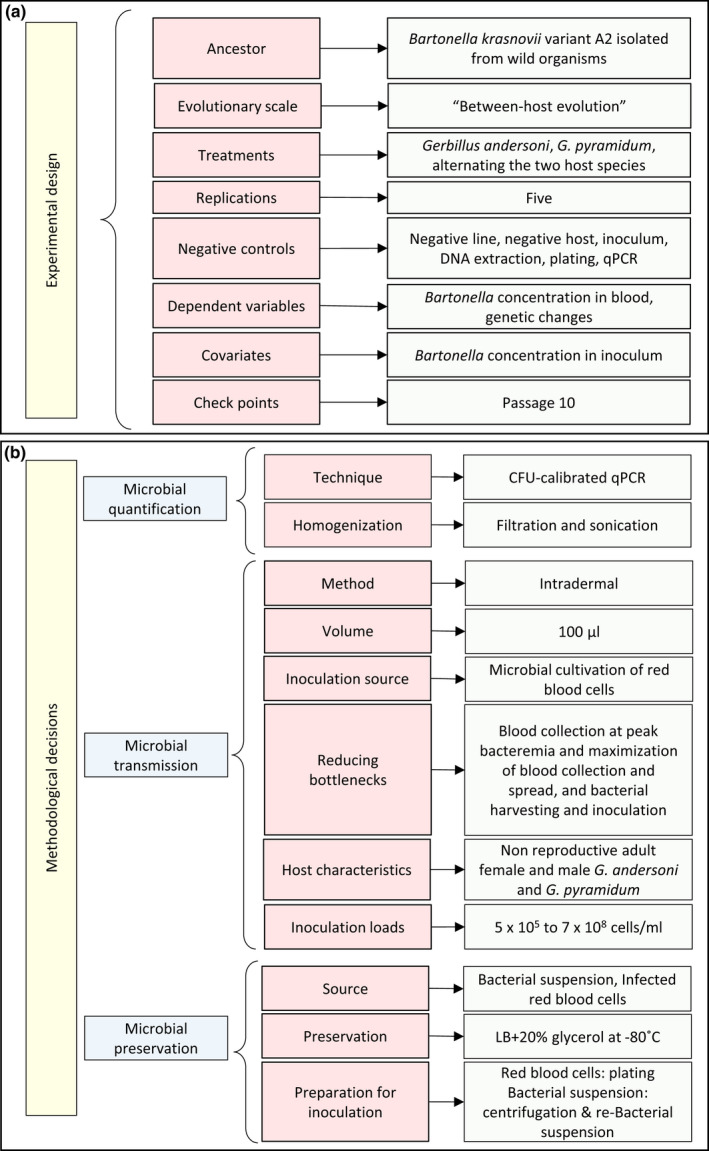
Summary of issues (red squares) and decisions (green squares) concerning experimental design (a) and specific methods (b) necessary to construct a protocol for an in vivo evolution experiment with blood‐borne microbial parasites. The final choices for our *Bartonella*‐wild rodent system are indicated in green, and they are based on the preliminary tests summarized in Table 2. CFU, colony forming units; qPCR, quantitative polymerase chain reaction; LB, lysogeny broth

To extend our knowledge of the evolutionary trajectories of blood‐borne parasitic microbes, and to apply this knowledge to predict and control the spread, outbreaks, emergence, and re‐emergence of diseases, it is crucial to design and then follow an appropriate protocol for any in vivo evolution experiment. In the sections that follow, we offer a road map that guides researchers through the decisions and preliminary experiments that are necessary for constructing an effective experimental protocol, one that matches the goals of their study and the natural history of their system (Figure 1). We use a *Bartonella*‐wild rodent system that we study as an example of applying this guide. The overall goal of this road map is to encourage researchers to design and perform effective in vivo evolution experiments with blood‐borne parasitic microbes, despite the challenges that they entail.

## 5 CASE STUDY OF A SEMINATURAL BACTERIUM‐RODENT (*BARTONELLA*‐*GERBILLUS*) SYSTEM

*Bartonella* infect rodents throughout the northwestern Negev Desert's sands in Israel. This system has several features that make it a good model to illustrate the use of our proposed road map. First, while rodents and their *Bartonella* parasites can be maintained and propagated in the laboratory under semi‐natural conditions, they represent a natural association, one in which the pathogens establish bacteremia (i.e., bacteria in the circulating blood) without significantly harming their natural hosts (Eidelman et al., 2019). Thus, this system reflects the challenges of working with natural hosts and bacteria, including potential difficulties of growing, isolating, quantifying, and marking the microbes, determining their generation time in vivo, and manipulating their hosts. Second, *Bartonella* can be transmitted both within and between rodent species, and this transmission occurs mainly through fleas (Morick et al., 2011). Thus, this system is representative of a major group of blood‐borne parasitic microbes that are transmitted by arthropod vectors. Working with vector‐borne microbes adds another complication for in vivo evolution experiments, because one must either include the vectors in the host‐to‐host transmission process or perform laboratory procedures that emulate the transmission by vectors (e.g., Riemersma et al., 2021). In any case, studying vector‐borne microbes offers an opportunity to gain insights into how evolution proceeds when parasites are propagated through multiple host types (i.e., the vector and the host). Also, by ascertaining the vectors' natural loads, one may achieve a good approximation for the inoculum volumes and concentrations that characterize transmission in nature.

Third, although *Bartonella* bacteria were previously transferred between host individuals for other proposes (e.g., studying the pathogenesis of the bacteria and the host immune response; Regnath et al., 1998), and although they have been used for in vitro evolution experiments (Gutiérrez, Markus, et al., 2018; Meghari et al., 2006; Werner et al., 2007), there is no published protocol for performing in vivo evolution experiments with *Bartonella*. We hope that establishing such a protocol will sow the seeds toward improving our knowledge of how this diverse and widely distributed genus—one that includes emerging and re‐emerging pathogens—evolves and adapts to natural hosts. Moreover, this system may help shed light on the potential of *Bartonella* species as zoonotic pathogens, and on the mechanisms responsible for the remarkable diversity of this genus in natural communities (Gutiérrez, Cohen, et al., 2018).

All protocols related to the rodents and *Bartonella* bacteria, as well as the relevant permission numbers approved by the IACUC, can be found in the Appendix S1.

## 6 A ROAD MAP OF DECISIONS FOR DESIGNING IN VIVO EVOLUTION EXPERIMENTS WITH BLOOD‐BORNE PARASITIC MICROBES

### 6.1 Experimental design considerations

Before starting an in vivo evolution experiment, one must choose the host and starting microbial genotypes as well as the evolutionary scale of interest (within‐ or between‐host evolution). One must also decide on the treatments, the number of replicates per treatment, and the type and number of negative controls, as well as identify the dependent variables, covariates, and check points (Figure 1a).

In vivo serial passage experiments with microbes are often derived from a single ancestral clone. Thus, all genetic differences that evolve result from new mutations that occur independently in different replicates (Lenski, 2017a). This approach facilitates comparisons between the sets of populations evolving from the common ancestor under different treatments, and it removes intergenotype competition at the beginning of the experiment, thereby simplifying the experimental set up. Moreover, by minimizing the within‐group variability, it increases the statistical power to detect significant differences among treatments. An alternative approach would be to begin some replicate lines with different ancestor genotypes. This approach would reduce the number of replicates available for studying each individual genotype (assuming limited experimental resources), but it could provide a more general, less genotype‐dependent perspective on the study goals and results (e.g., Mackinnon & Read, 1999). For some proposes, such as emulating the level of genetic diversity in nature, tracking the emergence and spread of mutants, or under specific practical constraints (e.g., the microbe is nonculturable and can be transmitted only by blood‐to‐blood inoculations), yet another approach would be to begin with a single ancestral population that already possesses some genetic variation. This variation could be achieved by initializing all replicate lines with a single parasite‐positive blood sample containing diverse genotypes, or by artificially mixing multiple known genotypes (e.g., de Roode et al., 2005).

The collection of ancestral genotypes used to initiate an evolution experiment could include standard laboratory strains, recently isolated strains that have been passaged only a few times in the laboratory, or natural isolates that are taken directly from hosts, depending on the study goals and constraints. When the ancestral parasite can be isolated from various vector or host groups (e.g., from different host species, sexes, and genotypes), or directly from the environment, the match between the ancestor source and hosts used in the serial passages may strongly influence the inoculation and transmission success (e.g., Mackinnon & Read, 1999) and thereby also affect the evolutionary trajectories (e.g., Kubinak et al., 2015).

Microbes can evolve within a single host organism during the course of an infection, as well as more gradually as they infect and are transmitted between multiple host individuals. The evolutionary scale of a study should be determined based on its goals. Within‐host evolution is especially likely to occur in parasites of long‐lived hosts, presuming the parasites are able to persist inside one host for long periods. Examples of within‐host evolution include *Mycobacterium tuberculosis*, *Staphylococcus epidermidis*, *Pseudomonas aeruginosa*, and human immunodeficiency virus in humans (Genestet et al., 2021; Marvig et al., 2014; Poon et al., 2010; Zhou et al., 2020), H5N1 influenza viruses in humans and poultry (Moncla et al., 2020), foot‐and‐mouth disease virus in cattle (Fish et al., 2020), *Salmonella enterica* in mice (Diard & Hardt, 2017), and tobacco etch potyvirus in plants (Cuevas et al., 2015). The opportunity for within‐host evolution is limited for parasites with short infection periods; thus, one typically allows such parasites to evolve over the course of transmission through multiple host individuals (Moncla et al., 2020). In vivo investigations of within‐host evolution often rely on latitudinal sampling of individual hosts, followed by genomic analyses of the target microbes. In studies at this evolutionary scale, the methodological decisions related to microbial transmission between hosts are not relevant. While it is beyond the scope of this review, reviews on this topic can be found elsewhere (Culyba & Van Tyne, 2021; Lauring, 2020).

Any population may evolve through random genetic changes as well as by adaptation to selective conditions in an experiment. Therefore, most evolution experiments include at least two treatments, one of which serves as a control that is maintained under the same conditions as the experimental group, but which does not experience the treatment in question. For example, one might examine the evolution of parasites in hosts that are undergoing some therapy for the infection; in that case, one might include a control in which parasites evolve in untreated hosts. In vivo experiments with blood‐borne microbes also require another type of negative control, namely one or more hosts that are inoculated using the normal procedures but without including the microbe. A negative control “line” that is transmitted from one clean (i.e., uninfected) host to the next throughout an experiment can help detect issues with antiseptic work and cross‐contamination. However, if the negative host becomes contaminated, it might be difficult to determine the source and timing of contamination, with possibilities including overall contamination (e.g., in the media), a localized problem (e.g., cross‐contamination between sequential hosts), or a recipient host that was infected before the sham inoculation. Therefore, we recommend having multiple negative controls, including a negative control “line” and, during each passage, specific negative controls for each procedure that should be saved and tested (e.g., inoculation, cultivation, DNA extraction, and quantitative polymerase chain reaction; qPCR). To distinguish genetic changes that are favoured during in vivo evolution from those enriched during in vitro culturing steps, we recommend adding another control type, in which the microbes are passaged wholly in vitro. However, the generation time of the microbes is likely to differ in the two environments (in vivo and in vitro). Therefore, this approach typically cannot be used for quantitative comparisons of the rates at which mutations accumulate, but it can still be useful for determining whether particular genetic changes are associated with a specific environment.

The choice of dependent variables, covariates, and check points depends on a study's goals, the natural history of the host–parasite combination, and one's knowledge of the study system. The dependent variables will probably include quantifying genetic (e.g., rate of mutations and genetic variation; Jerzak et al., 2007) and phenotypic changes (e.g., growth rates and relative fitness; Coffey et al., 2008). Phenotypic changes may also sometimes relate to experimental procedures; for example, the inoculation load itself might evolve (Ciota et al., 2009). Covariates may relate to the inoculation process (e.g., the inoculation volume; Marignac et al., 2010), or they may describe specific host traits (e.g., host gender, genotype, species, or previous experience; Jerzak et al., 2008) and environmental conditions (e.g., temperature). Finally, it is important to set some checkpoints along the multiple passages to ensure there has been no contamination and to determine whether genetic changes are occurring. The checkpoints should reflect one's understanding of the microbe's generation time and evolutionary rate as well as the expected duration of the study.

**FIGURE 2:**
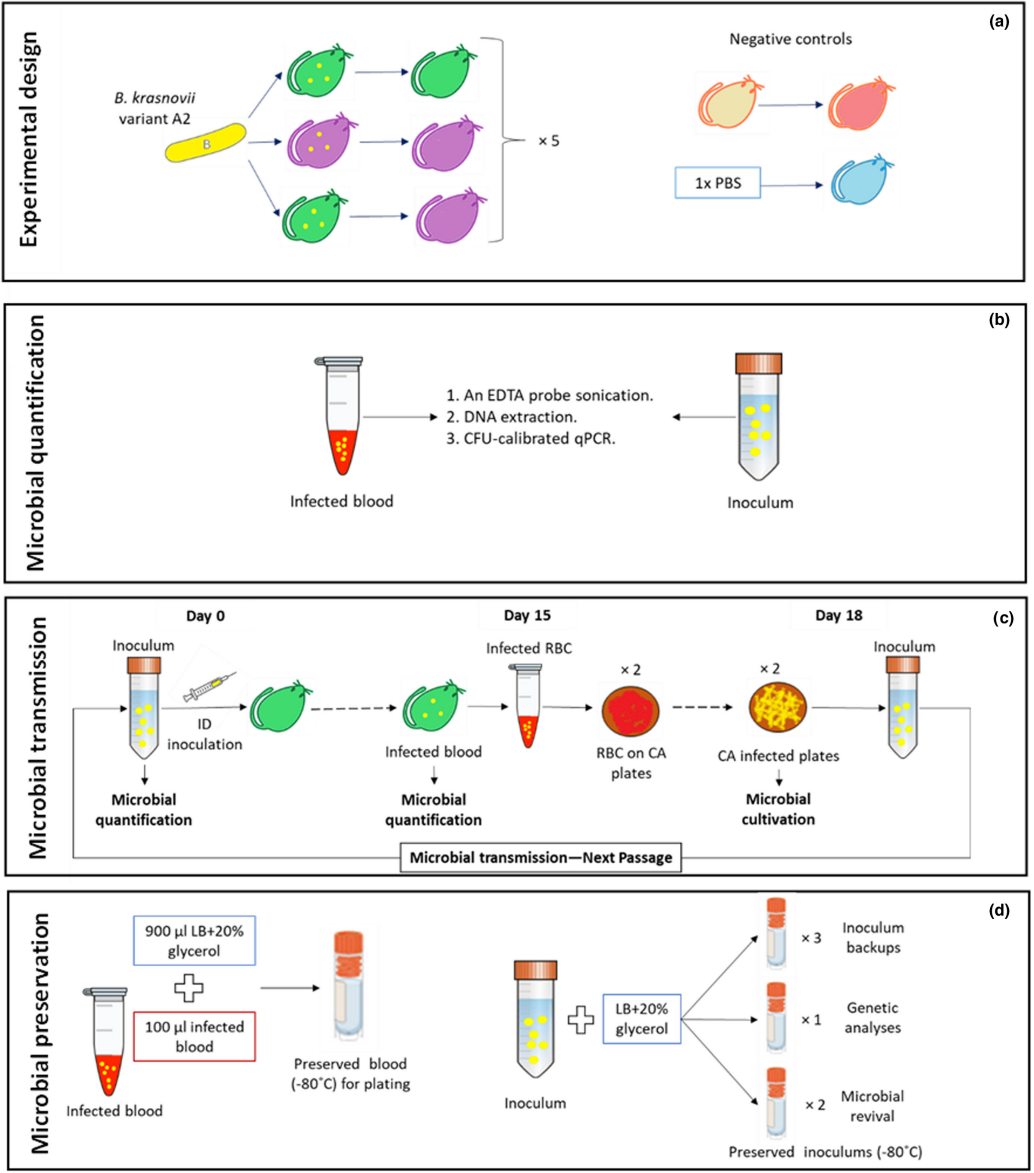
Schematic illustration of the full protocol for our in vivo evolution experiment. Illustration of the steps in the protocol that were chosen based on the preliminary results (Table 2 and Figure 3). (a) Overall experimental design. (b) Methods for microbial quantification. (c) Full set of procedures for microbial transmission in each serial passage. (d) Methods for microbial preservation. Green and purple shapes represent *Gerbillus andersoni* (GA) and *G. pyramidum* (GP), respectively; orange shapes show uninfected control animals of both species; and the blue shape shows a PBS‐inoculated control rodent. Yellow dots indicate bacterial infections in animals or bacterial cells in either blood or inoculum samples. EDTA, ethylenediaminetetraacetic acid; CFU, colony forming units; qPCR, quantitative polymerase chain reaction; ID, intradermal; RBC, red blood cells; CA, chocolate agar plate; LB, lysogeny broth. In C, only GA hosts are shown for simplicity

The overarching goal of our evolution experiment with the *Bartonella*‐rodent system is to quantify the effect of host‐species heterogeneity on the evolution of genetic diversity in the parasite. Our motivation is the remarkable diversity of *Bartonella* observed in natural communities (Gutiérrez, Cohen, et al., 2018). Thus, we chose to use a natural isolate as the ancestor. More specifically, we used an isolate of *B. krasnovii* variant A2 because it represents the most common lineage infecting the two most abundant rodent species in the study system, *Gerbillus andersoni* and *G. pyramidum* (80% and 60% prevalence in blood samples collected from these two host species, respectively; Gutiérrez, Cohen, et al., 2018). This variant was also previously used as the ancestor for an in vitro evolution experiment (Gutiérrez, Markus, et al., 2018), its genome has been fully sequenced (NCBI GenBank accession CP031844; Gutiérrez, Cohen, et al., 2018), and it was successfully inoculated into *G. andersoni* hosts under laboratory conditions (Eidelman et al., 2019). For simplicity, we decided to start all replicate lines with the same ancestor clone. This clone was originally isolated from *G. andersoni* blood. This choice allowed us to explore the evolutionary potential of wild‐type bacteria, while minimizing the number of replications in our first in vivo evolution experiment. We did not expect that *Bartonella*—a slow growing, limited‐term pathogen (Eidelman et al., 2019)—would show much evolution during an infection of a single host. Therefore, we are examining evolution over multiple host infection and transmission cycles. We designed our experiment with three treatments: two homogeneous environments in which the bacteria are passaged through individuals of a single host species (*G. andersoni* or *G. pyramidum*), and a heterogeneous environment in which the bacteria are transmitted through individuals of the two host species, alternating at each passage. The experiment is constrained by the fact that we can simultaneously have a maximum of 17 individual rodents. Therefore, we included in the experiment five replicate lines per treatment, a negative control “line”, and a negative control host inoculated with phosphate‐buffered saline (PBS) (Figure 2a). The constraints on our study also did not allow us to include in vitro controls, which would require the plating and counting of multiple samples every 3 days. However, our previous in vitro evolution experiment with the same ancestral genotype of *B. krasnovii* found that, on average, only one mutation had accumulated in the experimental lines after 1000 bacterial generations (Gutiérrez, Markus, et al., 2018). Thus, without the host selection pressure, there was little opportunity for mutations to accumulate under these conditions. Therefore, instead of using in vitro control lines to examine whether the genetic changes that occur are adaptive to the host environment, we plan to compare the fitness of the evolved genotypes relative to the ancestral genotype, using both in vitro and in vivo environments, by performing competition experiments after the evolution experiment is complete.

Considering our study's goal, the dependent variables are the concentration of *Bartonella* cells in the host blood at each passage and the number of unique and parallel (i.e., common to multiple lines) genetic changes over time. The inoculum concentration serves as a covariate. We have planned to run the experiment for a total of 20 passages, and the tenth passage will serve as a checkpoint.

### 6.2 Methodological considerations

The challenges that are associated with in vivo experiments in blood‐borne parasitic microbes (see section 3) require careful consideration of the procedures used for microbial quantification, transmission, and preservation (Figure 1b). Although mostly technical, these issues also often have conceptual implications, as discussed below.

#### 6.2.1 Microbial quantification

##### 6.2.1.1 Quantification technique

Microbial quantification is required for assessing inoculation success before and during the evolution experiments, and for assessing changes in microbial load per host during the experiment, including differences among treatment groups that may result from parasite adaptation. Plating procedures that count colony‐forming units (CFUs) or plaque‐forming units (PFUs) are conventional techniques that are often used to estimate the number of viable cells or viruses for culturable microbes. At the other extreme, PCR, qPCR, and droplet digital PCR (ddPCR) are molecular techniques that are used to detect and quantify microbial DNA, without requiring that the microbes be culturable, and without distinguishing between live and dead cells or intact and inactivated viruses. Flow cytometry (FCM) and flow virometry (FVM), as well as various types of microscopy (e.g., with and without staining), can be used to classify and quantify microbes based on their morphological characteristics, again without the need for cultivation, and these approaches can be designed to distinguish between live and dead cells (Ou et al., 2017).

**FIGURE 3:**
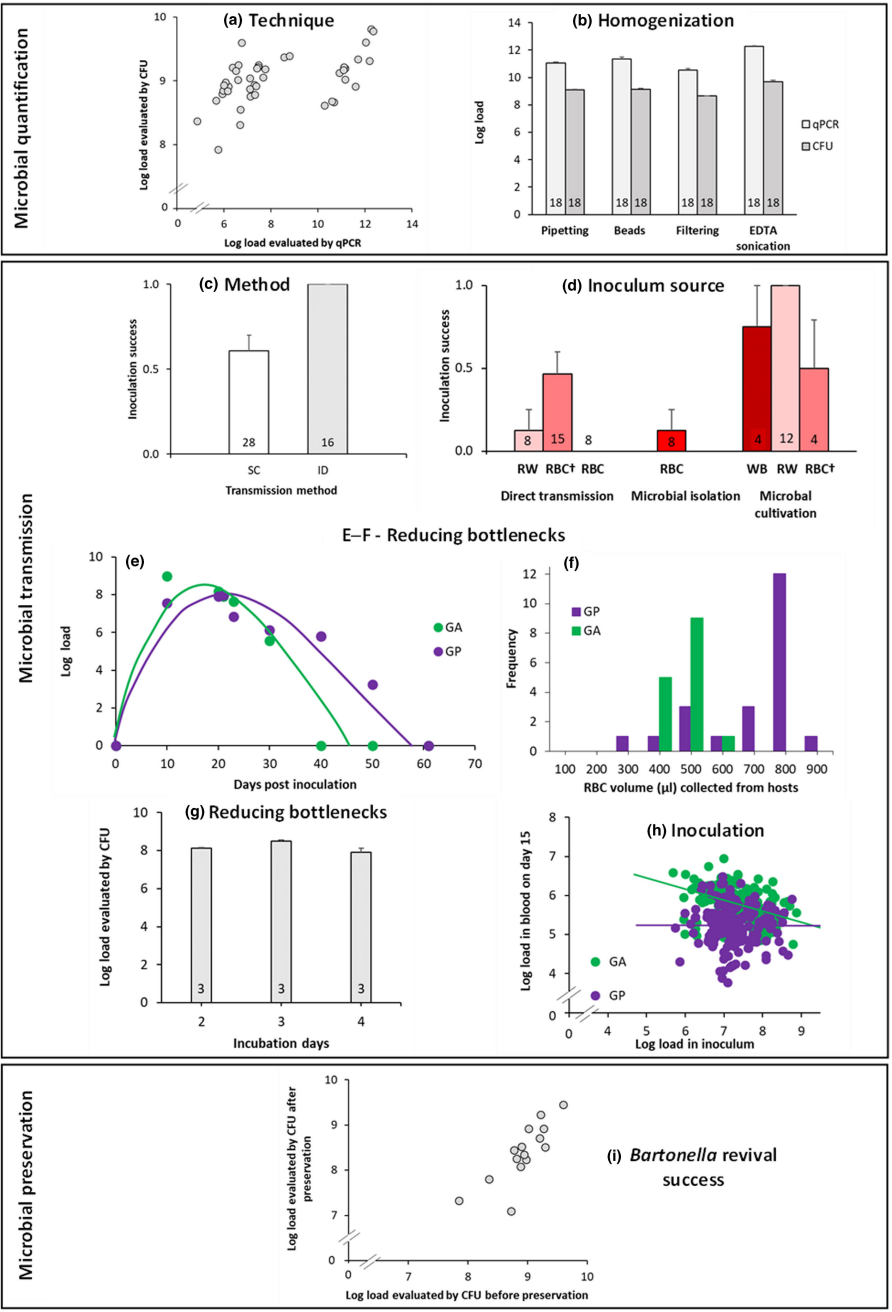
Preliminary results used to select the methods for our in vivo evolution experiment. Results are shown for the various steps used in microbial quantification (a,b), transmission (c–h) and preservation (i). Bar plots represent population means, with sample sizes shown therein, and standard errors. *Bartonella* loads are given per ml, and they were estimated by quantitative polymerase chain reaction (qPCR) unless indicated otherwise. SC, subcutaneous inoculation; ID, intradermal inoculation; EDTA, ethylenediaminetetraacetic acid; WB, whole blood; RW, red and white blood cells after plasma removal; RBC, red blood cells; RBC†, RBC after density gradient centrifugation (DGC) with Ficoll; WBC, white blood cells; GA, *G. andersoni*; GP, *G. pyramidum*; CFU, colony forming units Curves in Figure 3e are intended to aid visualization; they are not fit to the data points

Table 1 compares the properties of the most common detection and quantification techniques. The technique of choice ultimately depends on the research goals (e.g., the importance of estimating the number of live cells), study system (e.g., whether the organisms are culturable and, if so, their generation time), and practical aspects (e.g., equipment availability). When possible, we recommend using multiple techniques. After we failed to develop a reliable FCM protocol that works on blood samples and finding the CFU technique prohibitively time‐consuming for our slow‐growing microbe, we decided to quantify the inoculum and blood samples by qPCR (Figure 2b). To relate qPCR values to live *Bartonella* cell counts, we calibrated the qPCR assays using CFU counts. To validate the qPCR assays, we ran a preliminary experiment, in which the *Bartonella* loads in suspensions were simultaneously evaluated by the calibrated‐qPCR and CFU assays, and we observed a strong correlation (Table 2 and Figure 3a).

**TABLE 1:** Microbial detection and quantification techniques

	CFU or PFU	PCR	qPCR or RT‐qPCR	ddPCR	FCM or FVM
Sample source	Microbial cells or virus particles	DNA	DNA or RNA	DNA	Microbial cells or virus particles
Technique	Cell plating and counting	DNA amplification and product visualization by gel electrophoresis	DNA or RNA amplification, during which changes in fluorescent dyes that intercalate with DNA or RNA are quantified	Direct count of nucleic acid molecules	Individual cells flow through a laser beam and events causing light scattering are quantified
Detection or quantification	Both	Detection	Both	Both	Both
Need for cultivation	Yes	No	No	No	No
Sample state	Solid media	NA	NA	NA	Liquid media
Live and dead distinction	Counts only live cells or infectious particles	No	No	No	Sometimes, with appropriate calibration
Sensitivity	Low	Low	High	Very high	High
Time demand	Depends on the microbe's growth rate	High^a^	High^a^	High^a^	Low
Process time	Slow	Fast	Fast	Fast	Very fast
Potential biases	Cultivation media	Primer and probe affinity, DNA quality, inhibition by carryover of some molecules, enzyme inefficiency	Primer and probe affinity, DNA or RNA quality, reverse transcriptase quality, inhibition by carryovers of some molecules, enzyme inefficiency	DNA quality, narrow dynamic range	Quantification of nonspecific particles
Methodology considerations	Incubation time and conditions, dilution range	Primers and reaction conditions	Primers, probe, and reaction conditions, standard curve calibration	Primers, probe, dynamic range	Ability to distinguish between specific and nonspecific particles

*Note*: Comparison of properties among several common techniques.

Abbreviations: CFU, colony‐forming unit used for microbial quantification; ddPCR, droplet digital PCR; FCM, flow cytometry used for cell characterization and quantification; FVM, flow virometry used for viral characterization and quantification; NA, not applicable; PCR, polymerase chain reaction; PFU, a plaque‐forming unit used for viral quantification; qPCR, quantitative PCR; RT‐qPCR, reverse transcription quantitative PCR.

^a^ Including DNA extraction.

**TABLE 2:** Preliminary tests used to determine methodology for our in vivo evolution experiment

Process	Question		Results
Microbial quantification	Are CFU and qPCR values well correlated?		Yes ^a^ *R* ^2^ = .64; *p* < .001; Figure 3a
	How to achieve the highest *Bartonella* yield? After processing the suspension with pipetting (P), beads (B), 5‐μm filtering (F), or sonication (S)?		Highest after sonication ^b^ *F* = 75.31; *p* < .001; Figure 3b The differences between S and each of the other treatments are significant^c^
Microbial transmission	Method: Are there differences in the bacterial dynamics after SC versus ID inoculation?	Peak day	No differences ^b^ *F* = 0.75; *p* = .39
		Peak load	No differences ^b^ *F* = 2.2; *p* = .15
		First day as positive	No differences. ^b^ *F* = 0.02; *p* = .88
		Inoculation success	Higher success with ID. ^d^χ^2^ = 11.96; df = 1, *p* < .001; Figure 3c
		Inoculation load	Higher load with ID ^b^ *F* = 4.52; *p* < .05
	Inoculation source: Do the various sources differ in their inoculation success?	Rodents infected by direct blood transmission, or after microbial isolation or cultivation	Highest (100%) inoculation success with *Bartonella* cultivated from RW ^b^ *F* = 8.76; *p* < .001; Figure 3d The differences between microbial cultivation from RW and (i) direct RW, (ii) direct RBC†, (iii) direct RBC, and (iv) isolation from RBC are significant^c^
		In which blood fraction do *Bartonella* cells occur?	Almost exclusively in RBC Sample 1: 100% in RBC Sample 2: 100% in RBC Sample 3: 96% in RBC, 4% in WBC Sample 4: 100% in RBC
	Reducing bottleneck effects	When is the mean peak bacteraemia?	Between days 10–20 post inoculation Figure 3e
		What is the maximum RBC volume that can be sampled from most host individuals?	350 μL Figure 3f
		For how long should cell lawns be incubated to maximize the number of live bacteria?	Three days ^b^ *F* = 12.07; *p* < .01; Figure 3g The differences between 3 days and each of the other days (2 and 4) are significant^c^
	Inoculation load	Is there a threshold for inoculation success?	No threshold was detected for the inoculation loads that were tested
		Are inoculation load and infection success positively correlated?	Low negative correlation in GA No correlation in GP ^a^GA: *R* ^2^ = −.26, *p* < .001 ^a^GP: *R* ^2^ = −.05, *p* = .5 Figure 3h
Microbial preservation	Viability	Can *Bartonella*‐infected blood and inoculum samples be preserved in LB + 20% glycerol at ─80°C, then revived and inoculated into rodents?	Yes All 10 rodents that were inoculated with revived *Bartonella* from the two types of preserved stocks (6 and 4 samples, respectively) became infected
	Revival success	Are the bacterial loads of fresh and long‐term preserved inocula positively correlated?	Yes ^a^ *R* ^2^ = .88, *p* < .001 Figure 3i

*Note*: Results are provided according to the process and the corresponding question. When relevant, the results include the statistical tests and significance levels, and the corresponding figure is indicated. RBC†, RBC after density gradient centrifugation (DGC) with Ficoll. Direct RW, RBC†, or RBC, Blood components inoculated directly to the host rather than culturing or isolating the microbe beforehand.

Abbreviations: CFU, colony‐forming unit; GA, *G. andersoni*; GP, *G. pyramidum*; ID, intradermal inoculation; LB, Lysogeny broth; qPCR, quantitative polymerase chain reaction; RBC, red blood cells; RW, red and white blood cells after plasma removal; SC, subcutaneous inoculation; WBC, white blood cells.

^a^ Pearson's correlation test.

^b^ One‐way ANOVA.

^c^ Tukey–Kramer post hoc test.

^d^ Generalized linear model with binomial distribution and logit link function.

##### 6.2.1.2 Homogenization technique

Regardless of the quantification techniques that are employed, it is important to avoid microbial aggregation, as aggregates reduce the precision and repeatability of quantification (Trunk et al., 2018). To address this issue in our study, we compared the estimated load of *Bartonella* cells in suspensions subjected to the following procedures: (i) pipetting; (ii) mechanical separation using glass beads and vortexing; (iii) mechanical separation using glass beads, vortexing, and 5‐μm filtration; and (iv) treatment with 0.25 M Ethylenediaminetetraacetic acid (EDTA), which inhibits the adhesion of bacteria, followed by probe sonication. We found that probe sonication resulted in the highest CFU counts (Table 2 and Figure 3b). Accordingly, we decided to sonicate the cells before qPCR and CFU quantification (Figure 2b).

#### 6.2.2 Microbial transmission

##### 6.2.2.1 Transmission method

There are various methods for transferring blood‐borne microbes between hosts in the laboratory including vector‐mediated, subcutaneous (SC), intramuscular, intraperitoneal, intradermal (ID), intravenous, and intraocular inoculations. These methods range from ones that require little or no human intervention, such as transmission by arthropod vectors (e.g., Bellone et al., 2020), to others that require increasing levels of training and dexterity while providing greater control, from basic subcutaneous (e.g., Michelitsch et al., 2021) and intramuscular (e.g., Bastos et al., 2020) inoculations to more advanced methods including intraperitoneal (e.g., Kosoy et al., 1999), intradermal (e.g., Conlan et al., 2003), intravenous (e.g., Marignac et al., 2010), and intraocular inoculations (e.g., Marignac et al., 2010). The choice of the method will probably affect the rate of successful transmission as well as the potential for transmission bottlenecks, depending on the microbe's characteristics and natural transmission routes. The choice of the transmission method may also depend on the study goals and feasibility.

We attempted to establish a reliable procedure for transmitting *Bartonella* through fleas. However, the intended recipient rodents did not become infected when exposed to *Bartonella*‐infected fleas. Two caveats of using alternative methods are that they may not emulate the natural transmission route that the microbe encounters in nature, and that the microbial population will no longer be subjected to selection in the vector (Riemersma et al., 2021). To address the first caveat, we tested the most relevant alternative methods for arthropod‐borne microbes, namely SC and ID inoculations. ID inoculations resulted in significantly higher success rates and bacterial loads (Table 2 and Figure 3c). ID inoculations also better emulate flea‐borne transmission (Hong et al., 2017), and so we chose to use this method in our evolution experiment (Figure 2c). The second caveat cannot be fully solved, but the in vitro phases that we decided to include between the in vivo transmissions (see the section “Inoculation source”) may provide a rough approximation for how *Bartonella* proliferates in ectothermic vectors.

##### 6.2.2.2 Inoculum source

**FIGURE 4:**
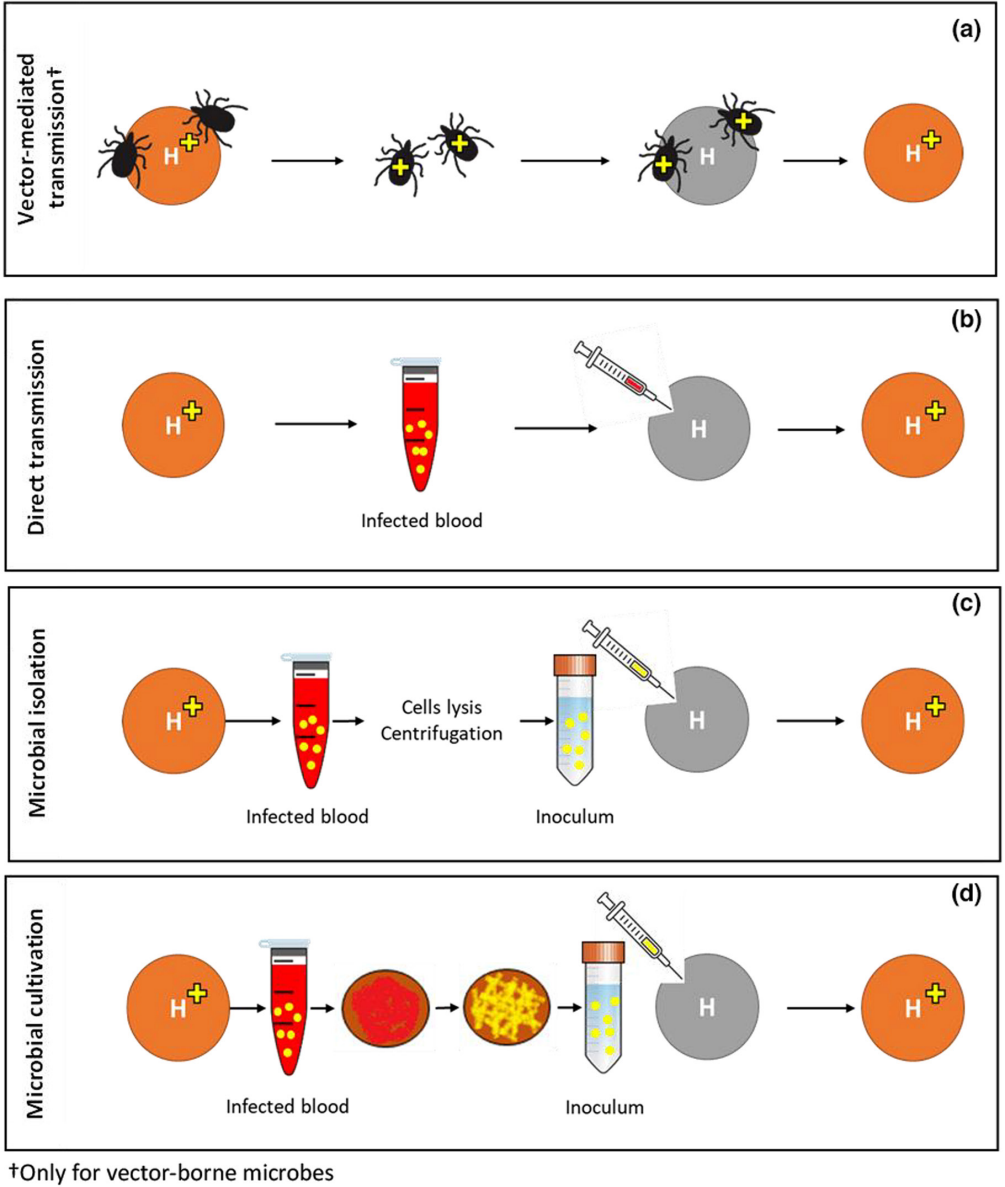
Alternative sources for blood‐borne microbial transmission in evolution experiments. Three alternative sources can be used for blood‐borne microbial transmission in evolution experiments, depending on study goals, parasite (yellow shapes) and host (circles) natural histories, background knowledge, and practical considerations. First, infected blood can be transmitted by vectors (black arthropods) that fed on infected hosts (orange circles) and are then moved to uninfected hosts (grey circles) (a), or by direct blood transmission, in which blood (or specific blood fractions) are drawn from infected hosts and inoculated into uninfected hosts (b). Second, microbes can be isolated from the blood of infected hosts and directly inoculated into uninfected hosts (c). Third, microbes taken from the blood of infected hosts can be cultivated, harvested, and inoculated into uninfected hosts (d). †Only for vector‐borne microbes

Several sources of the inoculum can be used for transmitting blood‐borne microbes between host individuals, including infected blood, isolated microbes, and cultivated microbes, which range from the most to the least natural scenarios (Figure 4). Transmission of infected blood (whether by direct transfer or mediated by a vector) emulates natural conditions, but it requires comprehensive knowledge of the study system and may unintentionally transmit other microorganisms or immune factors and cells (Figures 4a,b). Also, using infected blood as the source may sometimes fail to achieve adequate infection success under artificial conditions (Table 2 and Figure 3d), and it risks harming the host in ways that are unrelated to the study question, for example, due to the introduction of blood factors from a different host individual. An alternative approach is to lyse the blood cells of infected hosts, isolate the microbes, and inoculate the microbial suspension into uninfected hosts (Figure 4c). When it is necessary to control or increase the number of microbial cells in the inoculum, or if none of the alternative methods work, one can cultivate the microbes in the infected blood (or specific blood fractions) either in liquid media or on plates, then harvest the cells, resuspend them, and inoculate the microbes into uninfected hosts (Figure 4d). This strategy of microbial cultivation allows frequent checking for contamination and storage of intermediate steps; for vector‐borne microbes, it also allows some replication outside of the host, as might occur in a vector. However, it also extends the duration of each passage and may introduce additional, unintended selection that favours those genotypes that are better at growing in the in vitro environment.

Knowledge of the natural transmission route and the locations that a microbe colonizes in the host can inform the decision of which inoculation source to use. When considering the alternative sources for infected blood (Figure 4c,d), it is important to know whether the microbes tend to aggregate, and if so, to consider applying a homogenization technique (as discussed in the section on “microbial quantification”). Homogenization would, on the one hand, enhance the control of infection load; on the other hand, it may produce biases by interfering with the natural tendency of the microbes to aggregate. If the microbe is sensitive to sonication or chemicals used in isolation procedures (e.g., Ficoll, a hydrophilic polysaccharide used for density gradient centrifugation [DGC], or ammonium‐chloride‐potassium, a buffer used to lyse red blood cells), then this sensitivity may also limit the suitable choices for the inoculum source.

For *Bartonella*, we compared the success of infecting rodents by direct blood transmission, microbial isolation, and microbial cultivation. Microbial cultivation of infected blood after plasma removal achieved the highest inoculation success (Table 2 and “RW” in the right side of Figure 3d). We further verified that the plasma removal process, which is part of this protocol, did not reduce the number of *Bartonella* cells. Accordingly, using DGC, we split four infected blood samples into plasma, white blood cell (WBC), and red blood cell (RBC) fractions, extracted the DNA, and ran qPCR. We confirmed that *Bartonella* were mainly located in the RBC fraction, indicating that we would not lose too many cells by plasma and WBC removal (Table 2). In our planned in vivo evolution experiment, we will compare between conspecific and heterospecific bacterial transmissions, and so to avoid potential immunity‐mediated biases, we also decided to manually discard the buffy coat (the thin layer of WBCs mixed with platelets above the RBCs), thus cultivating microbial cells solely from infected RBCs on chocolate agar (CA) plates (Figure 2c). Considering the tendency of our *Bartonella* strain to aggregate (Riess et al., 2007) and the need to keep these bacterial cells alive for transfers, we decided to homogenize the inoculum by vortexing with glass beads and 5‐μm filtering.

##### 6.2.2.3 Population bottlenecks

From an evolutionary standpoint, it is usually desirable to avoid artificial population bottlenecks, because severe bottlenecks can prevent genetic adaptation to the treatment and instead promote random genetic drift by decreasing the effective population size (Barrick & Lenski, 2013; Izutsu et al., 2021; Wahl et al., 2002). To minimize the bottleneck effect and avoid biases introduced during transfers, one should determine the sensitive steps and design assays that will maximize the number and randomize the type of cells (e.g., different genotypes present in the previous host) that are propagated from one host to another during those steps. In general, the higher the number of cells during the bottleneck, the greater the power of natural selection to fix beneficial mutations and to minimize the accumulation of deleterious mutations by random genetic drift. We propose to aim for at least 1000 cells during each step for several reasons: (i) it is often achievable in practice; (ii) it seems likely that transmission events are not much larger in many natural infections; and (iii) it provides a reasonable balance between allowing beneficial mutations to survive and avoiding the random fixation of mutations with deleterious effects. It should be acknowledged, however, that points (ii) and (iii) will depend on the particular study system. For example, vector‐borne pathogens may typically experience less severe bottlenecks than those transmitted by aerosols; and the fixation of deleterious mutations will be more frequent and evolutionarily important in parasites with high mutation rates, including RNA viruses.

In our study system, two main steps are particularly sensitive to bottleneck effects. The first involves blood collection from infected hosts. To reduce the bottleneck effect during this step, we chose to collect the blood on day 15 post‐inoculation, which was within the range of days when bacterial titers peaked in both host species (Table 2 and Figure 3e). The rodents' antibody concentrations are also close to their peak levels at that time (Hawlena et al., unpublished data), which should exert strong selection on the bacteria. Moreover, we bled the hosts by cardiac puncture, which maximizes blood collection, and then culture 175 μl of infected RBCs on each of two CA plates, for a total of 350 μl of infected blood (day 15 in Figure 2c). This procedure should increase the transfer inoculum and thereby ameliorate the bottleneck effect. In preliminary trials, we found that despite variability in the blood volume that could be sampled from different individuals, at least 350 μl could be sampled from most of them (Table 2 and Figure 3f), allowing us to standardize sampling across host individuals and species.

The second sensitive step in our system is inoculum preparation. To ameliorate the bottleneck effect during this step, we chose to produce bacterial lawns over two CA plates and harvest all cells after 3 days of incubation (day 18 in Figure 2c). This three‐day period maximizes the numbers of live bacterial cells (Table 2 and Figure 3g). Collecting lawns, as opposed to isolated colonies, randomizes sampling, which increases the effective population size and thus reduces the bottleneck effect. This approach also reduces the chance that a genotype adapted to the in vitro culture condition would take over during this step. However, it may also increase the possibility of contamination, because *Bartonella* grows slower than some other bacteria and has to be cultured on nonselective media to avoid unintended evolution of antibiotic resistance. Also, using a genetic marker is currently not feasible in our model system. To this end, we decided to implement procedures to increase our ability to detect contamination, should it happen, during the evolutionary experiment. First, we plan to use multiple negative controls, including a negative control “line” and, during each passage, negative controls for the transmission and quantification procedures that will be saved and tested (see section ‘Experimental design considerations’). If contamination occurs, it is likely that it would be found in at least one of these controls. Second, in each plating event, we plan to scan the plates for suspicious colonies that do not have the typical *Bartonella* morphology (creamy‐white colonies with a rounded edge), and we will subject a few random colonies per plate to colony PCR using specific *Bartonella* primers. Finally, we plan to extract the DNA of each inoculum and each infected blood sample, to confirm that *Bartonella* is present in suitable numbers (6 × 10^3^ cells /ml or more; Figure 2b). To further reduce the effect of population bottlenecking, we maximized the inoculum volume, using the largest volume possible for intradermal injection in these rodent hosts, which is 0.1 ml for a single injection site (Morton et al., 2001).

##### 6.2.2.4 Host characteristics

Various host characteristics including the species, sex, age, reproductive status, genotype, and body conditions may affect transmission efficiency and infection duration, and they may also influence a parasite's evolutionary trajectory (Cornwall et al., 2018; Duneau et al., 2012). For example, Duneau et al. (2012) have shown that male and female hosts of *Daphnia magna* can exert different selection pressures on *Pasteuria ramose*, a parasite that causes host castration, especially in females, which leads to gigantism and increased numbers of the parasite. Host choice depends on the study goals, knowledge of the host's natural history (e.g., whether the sexes differ in susceptibility to infection), and practical considerations (e.g., the ease of obtaining enough host individuals with the relevant properties).

Given that our goal in the case study is to compare the evolutionary trajectories of bacteria evolving through either conspecific or heterospecific hosts, we required two distinct host species (Figure 2a). To avoid biases related to host age, reproductive status, and sex, we chose to use only nonreproductive adult rodents and to balance the number of males and females across the replicate lines (i.e., each line will be passaged through ~50% female hosts). Also, these rodents do not develop an infection after reinoculation (Eidelman et al., 2019), and so we can use each individual animal as a host only once, and we cannot use any hosts that have been previously infected. Such naïve rodents, born in the laboratory to *Bartonella*‐free parents, are kept routinely in our laboratory under flea‐free and *Bartonella*‐free conditions, and their *Bartonella*‐free status is confirmed by a qPCR test prior to using them in our experiment.

##### 6.2.2.5 Inoculation dose

The success and effects of host‐to‐host transmission are often density‐dependent. Higher microbial loads in the inoculum are likely to increase inoculation success. Higher loads may also increase the harm to the host (e.g., Mook‐Kanamori et al., 2012). Moreover, when the study involves multiple host groups, both the inoculation success and resulting damage may be group‐specific (Palinauskas et al., 2008). Changing the dose of parasitic bacteria can substantially change the rate of appearance and clinical manifestations of disease, so it is important to match these doses as closely as possible to the natural infection cycle (Gaunt et al., 1996).

To determine the threshold inoculum below which an infection cannot be (or rarely is) established, we inoculated individual animals of each rodent species with different bacterial loads ranging from 5 × 10^5^ to 7 × 10^8^ cells/ml. We found that all of the loads we tested worked well; therefore, if there is a threshold load for successful transmission, it appears to be below 10^5^ cells/ml (Table 2). Moreover, we observed no positive correlation between the inoculation dose and the bacterial load on day 15 post‐inoculation (Table 2; Figure 3h). Finally, previous experiments suggest that bacterial loads within this inoculation range do not harm the rodent hosts used in our study (Eidelman et al., 2019).

#### 6.2.3 Microbial preservation

Planning for the collection and preservation of microbial samples during an in vivo evolution experiment is important because cases of host loss, contamination, and infection failure can be overcome by restarting the problematic lines from recently preserved samples, rather than having to restart the entire experiment. The preserved samples also serve as a frozen “fossil record” that can be revived by researchers to compare organisms, both genetically and phenotypically, from different generations and treatments (Lenski & Travisano, 1994; Mackinnon et al., 2005). The frozen samples can also be used in ‘replay experiments’, where evolution is restarted from intermediate generations to test whether specific outcomes are repeatable or contingent on certain prior changes (Blount et al., 2008, 2018). It is thus crucial to plan the preservation timepoints, the number of copies to save at each point, and the method of preservation, and to ensure that the preserved samples are viable and can be revived, cultivated, inoculated, and extracted after long‐term storage.

In this case study, our decision to control for the plated volume of infected blood results in extra blood that we store as backups, and which we can use in case this cultivation step fails or becomes contaminated. In addition, our protocol of inoculum preparation through growth on agar plates results in extra bacterial cells, which we decided to preserve and allocate during each passage as follows: (i) three cryotubes for new animal inoculations, (ii) one cryotube for genetic analyses of evolved bacteria, and (iii) two cryotubes for bacterial revival (Figure 2d). One or more of these samples could also be used later as additional backups or for other experiments.

To ensure that the *Bartonella* in the preserved blood and inoculum preparations are viable, we intradermally inoculated six and four uninfected rodents with the frozen backups of blood and bacterial cells, respectively, and all animals became infected (Table 2). To quantify the revival success, we correlated the *Bartonella* loads of 15 inoculum preparations that were either fresh or revived after long‐term storage, and we found a high correlation with only moderate cell loss (~30%; Table 2 and Figure 3i). These results thus support the efficiency of the two methods for preserving *Bartonella* populations.

## 7 CONCLUSIONS

Evolution experiments are valuable for understanding evolutionary dynamics, mechanisms, and the interplay between ecology and evolution (Kawecki et al., 2012; Lenski, 2017b). Such experiments and their interpretation entail multiple technical and conceptual challenges, some of which can be addressed using recent genetic and molecular technologies (Brockhurst et al., 2011; Dettman et al., 2012). Among evolution experiments, in vivo experiments that use parasites and pathogens are particularly challenging. However, such experiments can provide results that most closely mirror what happens in nature for these organisms, which often have critical ecological roles as well as substantial evolutionary potential owing to their large populations and rapid generations. Our road map is designed to address the major challenges of in vivo experiments and realize their potential in the context of blood‐borne microbial parasites. We illuminate this road map by examining critical experimental design and methodological considerations made while we established an in vivo evolution experiment with *Bartonella* and two rodent host species. We hope that this guide will encourage other teams to design and perform further in vivo evolution experiments—ones that are practical yet statistically powerful, span multiple host passages, and reflect natural transmission and other processes relevant for blood‐borne parasitic microbes. We also hope that our road map will help future teams design their studies by learning from our experience.

## 8 AUTHOR CONTRIBUTIONS

Hadas Hawlena and Ruth Rodríguez‐Pastor conceptualized the manuscript. All authors designed the experiments. Ruth Rodríguez‐Pastor, Nadav Knossow, and Yarden Shafran performed the experiments. Ruth Rodríguez‐Pastor wrote the initial draft of the manuscript, and all other coauthors edited the initial draft. The project was coordinated by Hadas Hawlena.

10

## 11 BENEFIT‐SHARING STATEMENT

Benefits from this research accrue from the sharing of our data and results on public databases as described above.

## Supporting information


Appendix S1

## Data Availability

The data that support the findings of this study are openly available in FigShare at https://doi.org/10.6084/m9.figshare.16641139
