# Supplementary material for: A road map for in vivo evolution experiments with blood‐borne parasitic microbes
Source: Mol Ecol Resour. 2022 Jun 6;22(8):2843–59. doi: 10.1111/1755-0998.13649 (PMC9796859; doi:10.1111/1755-0998.13649)
Supplement: Supplementary file 1 — Appendix S1 [file MEN-22-2843-s001.docx]

**Supplemental Information for:**

**A road map for *in vivo* evolution experiments with blood-borne**

**parasitic microbes**

Ruth Rodríguez-Pastor, Yarden Shafran, Nadav Knossow, Ricardo Gutiérrez, Shimon Harrus, Luis

Zaman, Richard E. Lenski, Jeffrey E. Barrick, and Hadas Hawlena

**Rodent maintenance, blood sampling, and bacterial inoculations**

The rodent hosts used in all experiments were non-reproductive adults of *Gerbillus andersoni* and *G. pyramidum* from a laboratory colony maintained by Hadas Hawlena. This colony derives from wild rodents that were born and raised in the laboratory. The animals have never been exposed to ectoparasites or any *Bartonella* spp., nor have they received any drug treatment. Rodents were housed individually in 20 × 30 cm^2^ disinfected plastic cages with a 1 cm layer of autoclaved sand as substrate, under semi-natural conditions of 25 ± 1°C, photoperiod of 12D:12L, *ad libitum* millet seeds, and daily alfalfa leaves as a water source.

During the subcutaneous inoculation, we manually restrained the rodent and injected the inoculum into the loose skin on the back of the animal’s neck, using a 23G needle. The inoculum volumes ranged from 150 to 500 µl. For the intradermal inoculation, we held the rodent in a gas anesthesia induction chamber to briefly anesthetize it with isoflurane (TERRELL Isoflurane, USP, liquid for inhalation, Piramal Critical Care Inc., USA). Then, we removed this individual from the chamber and placed its head in a nose cone to keep it anesthetized during the injection process. We shaved the dorsolateral skin of the rodent and then injected 100 µl of inoculum a few mm into the skin, using a 30G needle. A successful inoculation resulted in the formation of a bleb. Immediately after all inoculations, we returned the rodent to its cage; we checked daily and confirmed there were no skin reactions.

To assess the rodent’s bacterial load, we bled the rodents through the retro‐orbital sinus under general anesthesia by inhalation of isoflurane in a gas anesthesia induction chamber. Once the individual was fully anesthetized, we placed it in lateral recumbency, and added a drop of local anesthesia (Localin, Fischer Pharmaceutical Labs, Tel Aviv, ISR) into its eye. We collected the blood using capillaries coated with 0.14% Ethylenediaminetetraacetic acid (EDTA) and stored it in EDTA blood collection tubes (Microvette, 500 µl, SARSTEDT Group) at −20°C for later molecular analyses.

The handling protocol was approved by the Committee for the Ethical Care and Use of Animals in Experiments of Ben-Gurion University of the Negev (permission numbers IL-12-09-2018B and IL-76-09-2019B), and the animals were held in the laboratory under the Israel Nature and Parks Authority permissions (numbers H2265/2018, H3871/2019, H5110/2020, and H5970/2021).

**Isolation, culture, and quantification of *Bartonella krasnovii***

*Bartonella krasnovii* variant A2 was isolated from the blood of a wild *G. andersoni* host, as described in Gutiérrez et al. (2018). We stored the bacterial stock in 1 ml of lysogeny broth supplemented with 20% glycerol at −80°C. To prepare an inoculum, we revived the frozen bacteria and isolated them by streaking three times for colonies on chocolate agar (CA) plates (Novamed, Jerusalem, ISR). At each isolation, we incubated the plates. We suspended the last isolated colonies in Phosphate-buffered saline (PBS) and spread the suspension on CA plates. To prepare the inoculum, we incubated the plates, then harvested and suspended the bacterial cells in PBS. All plates were incubated for three days at 37°C and constant 5% CO_2_. To confirm that the bacteria belong to the *Bartonella* genus, following Gutiérrez et al. (2018), we sampled the plates and subjected isolated colonies to Polymerase chain reaction (PCR), targeting an 800-bp fragment of the *gltA* gene, using PCR-ready high-specificity tubes preloaded with master mix (Synthezza Bioscience Ltd., Jerusalem, ISR). After amplification, we ran the PCR products on a 2% agarose gel, stained with ethidium bromide, and visualized them under UV light.

To culture infected blood from a *Bartonella*-positive animal, we collected the blood in EDTA tubes by cardiac puncture from euthanized rodents under deep terminal anesthesia, using a 23G needle. Then, we spread the infected whole blood or red blood cells on CA plates, sealed and incubated the plates, and harvested and suspended the bacterial cells in PBS to prepare an inoculum.

To assess the bacterial loads in the rodent blood, we extracted DNA from the blood samples using a QIAamp BiOstic Bacteremia DNA Kit (QIAGEN), following the manufacturer’s instructions. To assess the bacterial loads in the inoculum, we conducted a thermal DNA extraction (Gutiérrez et al., 2018). In each extraction session, we included a negative control, in which all the reagents were added to PBS without adding the blood or bacterial suspension.

We quantified the bacterial loads by real-time quantitative PCR (qPCR) (CFX Connect System, Hercules, CA, USA), using 2× qPCRBIO Fast qPCR Probe Blue Mix, Hi-ROX (PCR Biosystems), following Eidelman et al. (2019). We directly quantified colony-forming units by plating 5 µl of six 10-fold serial dilutions on two CA plates, followed by colony counting after three days of incubation.

**References**

Eidelman, A., Cohen, C., Navarro-Castilla, Á., Filler, S., Gutiérrez, R., Bar-Shira, E., Shahar, N., Garrido, M., Halle, S., Romach, Y., Barja, I., Tasker, S., Harrus, S., Friedman, A., & Hawlena, H. (2019). The dynamics between limited-term and lifelong coinfecting bacterial parasites in wild rodent hosts. *Journal of Experimental Biology, 222*, jeb203562. <http://doi.org/10.1242/jeb.203562>

Gutiérrez, R., Cohen, C., Flatau, R., Marcos-Hadad, E., Garrido, M., Halle, S., Nachum-Biala, Y., Covo, S., Hawlena, H., & Harrus, S. (2018). Untangling the knots: Co-infection and diversity of *Bartonella* from wild gerbils and their associated fleas. *Molecular Ecology, 27*(23), 4787–4807. <http://doi.org/10.1111/mec.14906>
